# Higher-order power harmonics of pulsed electrical stimulation modulates corticospinal contribution of peripheral nerve stimulation

**DOI:** 10.1038/srep43619

**Published:** 2017-03-03

**Authors:** Chiun-Fan Chen, Marom Bikson, Li-Wei Chou, Chunlei Shan, Niranjan Khadka, Wen-Shiang Chen, Felipe Fregni

**Affiliations:** 1 Spaulding Neuromodulation Center, Department of Physical Medicine & Rehabilitation, Spaulding Rehabilitation Hospital and Massachusetts General Hospital, Harvard Medical School, Boston, MA, USA; 2 Engineering Science, Loyola University Chicago, IL, USA; 3 Department of Biomedical Engineering, The City College of the City University of New York, NY, USA; 4 Department of Physical Therapy and Assistive Technologies, National Yang-Ming University, Taipei, Taiwan; 5 School of Rehabilitation Science, Shanghai University of Traditional Chinese Medicine, Shanghai, China; 6 Department of Physical Medicine and Rehabilitation, National Taiwan University College of Medicine and National Taiwan University Hospital, Taipei, Taiwan.

## Abstract

It is well established that electrical-stimulation frequency is crucial to determining the scale of induced neuromodulation, particularly when attempting to modulate corticospinal excitability. However, the modulatory effects of stimulation frequency are not only determined by its absolute value but also by other parameters such as power at harmonics. The stimulus pulse shape further influences parameters such as excitation threshold and fiber selectivity. The explicit role of the power in these harmonics in determining the outcome of stimulation has not previously been analyzed. In this study, we adopted an animal model of peripheral electrical stimulation that includes an amplitude-adapted pulse train which induces force enhancements with a corticospinal contribution. We report that the electrical-stimulation-induced force enhancements were correlated with the amplitude of stimulation power harmonics during the amplitude-adapted pulse train. In an exploratory analysis, different levels of correlation were observed between force enhancement and power harmonics of 20–80 Hz (*r* = 0.4247, *p* = 0.0243), 100–180 Hz (*r* = 0.5894, *p* = 0.0001), 200–280 Hz (*r* = 0.7002, *p* < 0.0001), 300–380 Hz (*r* = 0.7449, *p* < 0.0001), 400–480 Hz (*r* = 0.7906, *p* < 0.0001), 500–600 Hz (*r* = 0.7717, *p* < 0.0001), indicating a t*r*end of increasing correlation, specifically at higher order frequency power harmonics. This is a pilot, but important first demonstration that power at high order harmonics in the frequency spectrum of electrical stimulation pulses may contribute to neuromodulation, thus warrant explicit attention in therapy design and analysis.

Peripheral nerve stimulation is broadly applied in clinical research to investigate sensory or motor function and to try to accelerate recovery in neurological conditions and disorders^1^,^2^,^3^,^4^,^5^,^6^. Stimulation applications include the activation of denervated muscles^7^ and modulation of corticospinal functions^8^. The efficacy of stimulation is determined by waveform parameters that include stimulation pulse frequency, intensity, and duration^9^,^10^. Taken together, stimulation parameters determine a power spectrum in the frequency domain, but the power spectrum is conventionally considered secondary in design and interpretation. Here, we aim to explicitly correlate the power at different frequency bandwidths (e.g. power harmonics) with activated force profiles. We adopt a previously validated animal model of peripheral nerve stimulation with an established central nervous system (CNS)contribution^11^,^12^,^13^,^14^.

Functional electrical stimulation uses pulse train waveforms (as opposed to sinusoidal or direct current^15^,^16^). Investigation of stimulation waveform optimization confirms that stimulation pulse frequency is central by setting action potential response rates^13^,^17^,^18^,^19^; with other parameters such as pulse duration and shape (e.g. charge balanced, biphasic) influencing activation threshold and safety^20^. Notwithstanding a role for pulse frequency - such as high or low stimulation frequency induced different levels of pain reduction or force enhancement - we posit that the power at higher harmonic frequencies influences neuromodulation. Therefore, instead of considering only the stimulation frequency of the individual stimulation pulses, we analyzed the power spectrum of the train of stimulation pulses, with special attention to power at the harmonic frequencies (integer multiples of the fundamental frequency^21^).

Our specific hypothesis is that during a session of ongoing pulsed electrical stimulation, we expect to observe correlation between power at high order harmonics of the stimulation waveform and neurophysiological responses acquired during the stimulation. We adapted an established animal model of peripheral nerve stimulation that induced corticospinal modulation which appeared to show discernable pain reduction or muscle force enhancement during electrical stimulation^13^,^14^,^22^,^23^.

Specifically, to test whether harmonics of a stimulation burst have a direct effect on corticospinal modulation, we evaluated the muscle force enhancement induced by a specific stimulation train which comprised a burst of higher stimulation intensity. This animal model was based on several earlier studies^13^,^14^, in which additional force, supposedly originated from a central mechanism, was observed when the specialized stimulation train was applied transcutaneously to intact neural pathways. In those studies, the additional force diminished when neural pathways proximal to the stimulation site were blocked, indicating that afferent (sensory) nerve fibers played a major role in generating additional force (i.e., the extent of corticospinal contribution). A correlation between the extent of corticospinal contribution and amplitude at power harmonics of the stimulation train would thus illustrate the concept behind our novel hypothesis.

## Methods

### Animal Subjects

Eight male New Zealand White rabbits (14–22 months old, weighing 3.0–4.0 kg) were anesthetized with isoflurane (AERRANE, Baxter, Deerfield, IL) at regulated concentrations during the experiments. Up to 5% of isoflurane was provided during induction, approximately 1% during the initial process, followed by an increase of around 3% if the rabbit showed any sign of regaining consciousness. The rabbits under anesthesia were determined to have reached the desired level of unconsciousness once they ceased to have a voluntary reflex response to foot pinch. Surgical drapes were wrapped around the rabbit to maintain physiological temperature. We did not use preanesthetic agents prior to stimulating the target muscle in order to avoid adverse effects associated with their application. Experiments were conducted in accordance with Institution Guidelines and were approved under the Affidavit of Approval of Animal Use Protocol, College of Medicine and College of Public Health, National Taiwan University.

### Experimental Setup

Anesthetized rabbits were constrained in a seated position with Velcro straps on a custom-made base as demonstrated in an earlier study^24^. Stimulation induced muscle force was acquired from a force transducer (RX-10, AIKOH Engineering, Osaka, Japan) that was clamped to the custom-made base. Force signals were sampled at 400 Hz by the force transducer and acquired by a data acquisition device (USB-6008, National Instruments, Austin, TX). Data were stored and processed using LabVIEW (National Instruments, Austin, TX). Electrical stimulation was applied on prepared skin (fur shave and application of depilatories) by positioning the cathode and anode electrodes over the skin surface closest to the femoral nerve and the quadriceps of the left hind limb respectively. A 1.5 × 1.5-cm disposable self-adhesive and reusable disk-shaped (diameter = 5.5 cm) flexible electrode were used as the cathode and anode. The precise nerve stimulation site for cathode (i.e. location closest to the femoral nerve) is determined by referring to a dissection manual^25^ and using a dissected rabbit hind limb from another study. Twitch stimulations were performed at least 24 hours prior to the actual experiments on each rabbit to pinpoint the exact site of stimulation. The stimulation site is then marked with a tattoo to ensure accurate positioning of the adhesive electrode during the actual experiments. Electrodes were positioned with adequate care to avoid inadvertent stimulation of the sciatic nerve. Stimulation and pulse patterns were transmitted from waveform generators (33220A and 33210A, Agilent Technologies, Santa Clara, CA) to a custom-made current-controlled power amplifier. Cathode-first charge-balanced biphasic pulses were administered to each rabbit in order to achieve efficacious action while minimizing tissue damage^20^. Electric pulses with a predetermined current intensity and waveform were applied in these experiments to generate force profiles that would result in better resolution of force readings. Since we were unable to specify the maximum voluntary contraction of a rabbit, we applied electric stimulations with current intensity that would evoke approximately 10 N of force, which is a fairly large sub-maximal force for a rabbit quadriceps to generate^26^. By applying burst stimulations to the rabbits at a frequency of 20 Hz using the electrical stimulation setup with a current intensity of 22 mA (determined by increasing the current intensity stepwise from 10 mA to 30 mA), which would induce approximately10 N of force was applied as the reference current intensity. Voltage and current readings of the electrodes were monitored with an oscilloscope (DPO2014, Tektronix, Beaverton, OR) and an instrumentation amplifier (INA128, Texas Instruments, Dallas, TX). The oscilloscope (Tektronix firmware 1.25) was also utilized to generate frequency spectrum in real time (sampling rate: 1 GS/s).

### Experimental Procedure and Data Analysis

**Figure 1:**
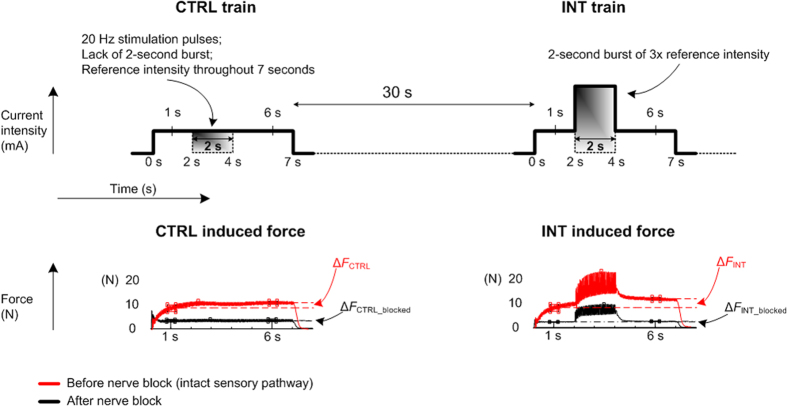
The electrical stimulation applied in this study include CTRL (control stimulation of reference intensity) and INT (intensity-modulated stimulation that includes a burst of increased intensity between 2 s and 4 s) trains that last 7 seconds. The profile of CTRL and INT induced force before and after nerve block demonstrates corticospinal contribution (Δ*F*_CTRL_ > Δ*F*_CTRL_blocked_ and Δ*F*_INT_ > Δ*F*_INT_blocked_) while the presence of increased intensity between 2 s and 4 s in unblocked/intact conditions demonstrates different levels of corticospinal contribution (Δ*F*_INT_ > Δ*F*_CTRL_).

In this study, two different types of intensity modulated stimulation trains of identical duration (7 s) comprising of biphasic electrical stimulation pulses (cathodic-delay-anodic: 250-62.5-250 μs) were applied at the same stimulation frequency (20 Hz) to each rabbit. As illustrated in the upper panels of Fig. 1, the first type of train is a control stimulation (CTRL) of reference intensity while the second type of train is an intensity-modulated stimulation (INT) that includes a burst of increased intensity between the 2 s and 4 s. The lower panels of Fig. 1 demonstrate the force profiles induced by CTRL and INT stimulation trains before and after blocking corticospinal contribution by injecting 6 ml of 20 mg/ml lidocaine and 0.06 ml of 1 mg/ml adrenaline proximal (region adjacent to the left L5-6 spinous processes) to the electrical stimulation site. The results were a replication of an earlier study^14^, demonstrating corticospinal contribution of peripheral nerve stimulation as the significant difference between the additional force acquired before and after nerve blocks (Δ*F*_CTRL_ > Δ*F*_CTRL_blocked_ and Δ*F*_INT_ > Δ*F*_INT_blocked_). The more additional force during the presence of increased intensity between 2 s and 4 s in unblocked/intact conditions (Δ*F*_INT_ > Δ*F*_CTRL_) suggested that a transient increase in stimulation intensity could modulate the amount of corticospinal contribution.

**Figure 2:**
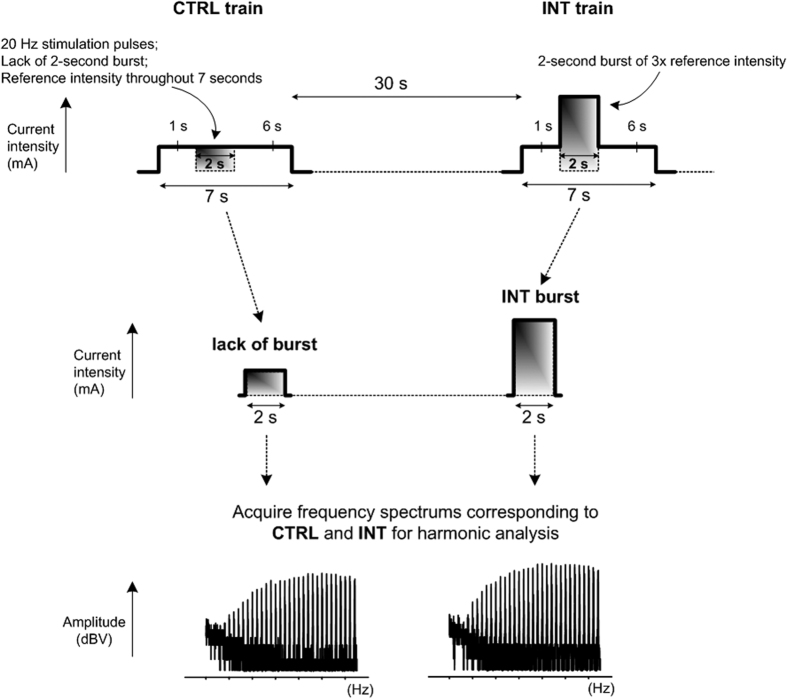
Frequency spectrums were acquired from the time interval between 2 s and 4 s of the CTRL and INT stimulation trains for harmonic analysis.

The objective of this study is to determine the correlation between the amount of corticospinal contribution and the power at the harmonics. We acquired the force profiles of the stimulation (lower panels of Fig. 1) and frequency spectrums from harmonic analysis between 2 s and 4 s of the stimulation trains (Fig. 2). Readings of the measured force from transducers were analyzed over 400 ms windows centered at 1 s and 6 s of each stimulation train. The amount of corticospinal contribution is determined by the normalized additional force (Δ*F*%), which is defined as “difference between the absolute force at 6 s (*F*_6s_) and the absolute force at 1 s (*F*_1s_), divided by the absolute force at 1 s (*F*_1s_) for normalization and expressed in percentage format 
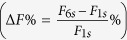
. Amplitude at harmonics of the stimulation frequency were extracted from frequency spectrums acquired spontaneously from the oscilloscope, wherein a Hanning Window was applied for better resolution with respect to frequency and magnitude^27^. The amount of corticospinal contribution (normalized additional force, Δ*F*%) and amplitude at the harmonics were correlated in terms of INT/CTRL for normalization (i.e., 
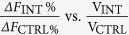
, where CTRL is considered the baseline of INT) to derive Pearson’s correlation coefficient (*r*). Paired t-tests were applied to the force profiles. Statistics were performed using Stata (StataCorp, College Station, TX) and *p* < 0.05 after Bonferroni Correction indicates a level of statistical significance. All data were expressed as mean ± SE.

## Results

### Force Profiles and Corresponding Frequency Spectrums

**Figure 3:**
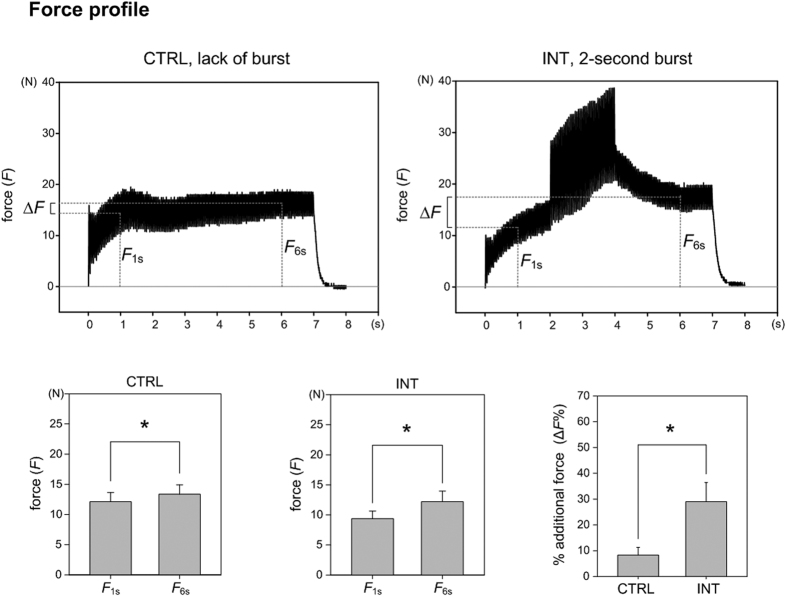
Force profiles corresponding to CTRL and INT stimulation trains of a single animal. In CTRL trains, force at 6 s (*F*_6s_) (13.36 ± 1.55N) was significantly greater (*p* = 0.0089) than that at 1 s (*F*_1s_) (12.11 ± 1.52N). In INT trains, *F*_6s_ (12.20 ± 1.76) was also significantly greater (*p* = 0.0035) than *F*_1s_ (9.37 ± 1.26). The normalized additional force is defined as Δ*F*% = (*F*_6s_ − *F*_1s_)/*F*_1s_% and comparison of normalized additional force (Δ*F*%) induced by the CTRL and INT trains indicate that Δ*F*% corresponding to INT trains (29.03 ± 7.39%) were significantly greater (*p* = 0.0107) than that corresponding to CTRL trains (8.31 ± 3.01%). *Indicates statistical significance (*p* < 0.05).

**Figure 4:**
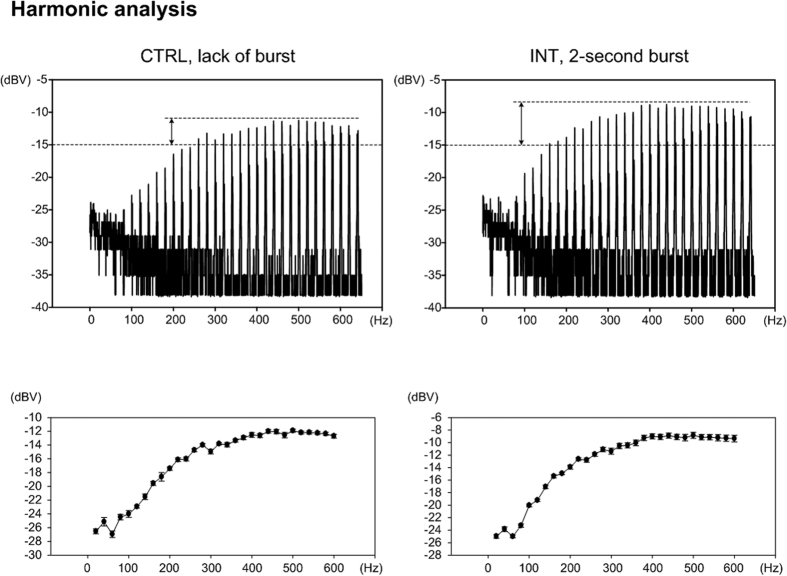
Harmonic analysis of frequency spectrums corresponding to CTRL and INT stimulation trains.

The force profiles (Fig. 3) of a rabbit and corresponding frequency spectrums of harmonic analysis (Fig. 4) demonstrate the variation of force and harmonics corresponding to CTRL and INT stimulation trains. In both CTRL and INT stimulation trains, *F*_6s_ was significantly greater than *F*_1s_. Hence, additional force (difference of the force at 1 s and 6 s indicated as Δ*F*) was observed in both CTRL and INT, wherein the stimulation intensity applied between 2 s and 4 s during the duration of the stimulation trains should be accounted for the additional extent of Δ*F*. The harmonics can be observed at integer multiples of the stimulation frequency (20 Hz) and is expressed in decibel units (dBV) to evaluate a wider range of different amplitudes. The bandwidth of frequency spectrums acquired were approximately 650 Hz, which covered the selective activation of certain afferent fibers that can be triggered either electrically^28^,^29^ or mechanically^30^ by sinusoidal signals.

### Correlating Additional Force (Corticospinal Contribution) with Power Harmonics

The Δ*F*% corresponding to INT (Fig. 4A) stimulation trains (29.03 ± 7.39%) were significantly greater (*p* = 0.0107) than that corresponding to CTRL trains (8.31 ± 3.01%) under paired-t tests. The dependent variables were continuous while the independent variables were matched pairs. By applying the Shapiro-Wilk test for normality, the results suggests that the data are approximately normally distributed (P_INT_ = 0.32549, P_CTRL_ = 0.12858; i.e., both failed to reject the null hypothesis that data is normally distributed). Based on an exploratory analysis of correlation between INT/CTRL ratios of Δ*F*% 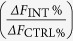 and harmonic amplitude 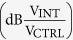 at the bandwidths of 20–80 Hz, 100–180 Hz, 200–280 Hz, 300–380 Hz, 400–480 Hz, and 500–600 Hz (Table 1), the 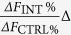 and 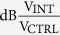 were more correlated at higher order harmonics (Table 2).

**Figure 5:**
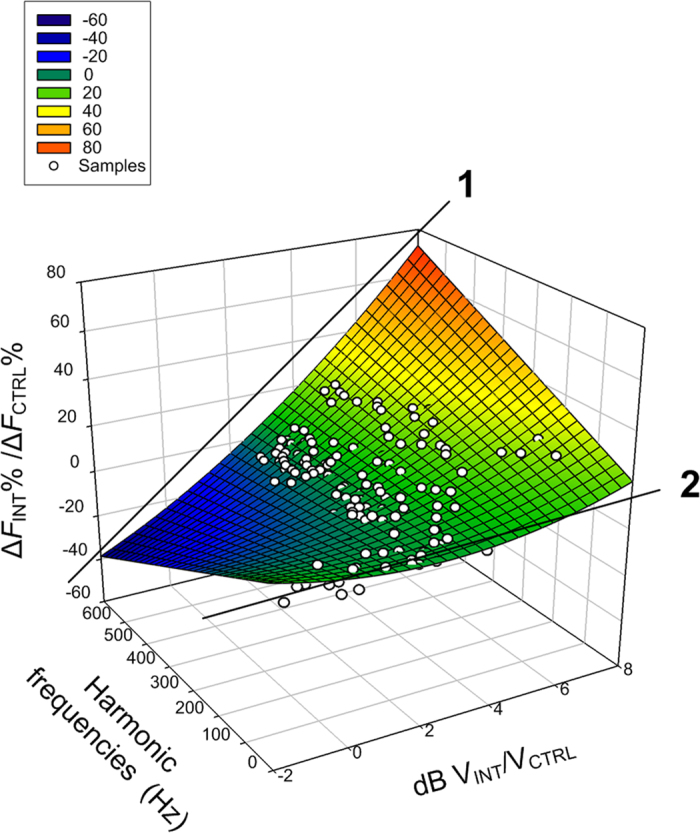
Samples (n = 240) of the INT/CTRL ratios at the stimulation frequency and harmonics (20–600 Hz) were fitted into a 3-D surface derived from a regression model. All coefficients of the regression model were significant (*p* < 0.0001). The colors correspond to the Δ*F*_INT_%/Δ*F*_CTRL_% axis. Line 1 indicates that higher correlation occurs at higher frequency whereas line 2 indicates that lower correlation occurs at lower frequency.

Samples (n = 240) of the INT/CTRL ratios 
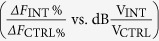
 at the stimulation frequency and harmonics (20–600 Hz) were plotted to fit into a 3-D surface (Fig. 5) derived from a regression model with SigmaPlot (Systat Software, San Jose, CA). All coefficients of the regression model were significant (*r*^*2*^ = 0.4224, *p* < 0.0001). Line 1 indicates that higher correlation occurs at higher frequency whereas line 2 indicates lower correlation at lower frequency.

## Discussion

In previous studies that attempted to induce force enhancement by peripheral nerve stimulation, Δ*F*% was observed only when the central nervous system was substantially communicating with the distal side of the extremity being stimulated^13^,^14^. It has been indicated that greater burst intensity, despite only occurring temporarily, resulted in an overall greater Δ*F*%^14^, which is consistent with the findings of this study. Based on the prior results, the increasing correlation between Δ*F*% and amplitude at high order harmonics indicate that the high order harmonics were probably the main factor that modulated the observed Δ*F*%. The significantly greater correlation between 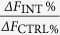 (normalized Δ*F*%) and 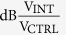 (normalized amplitude at harmonics) at higher order harmonics of the stimulation frequency supports our hypothesis that the amplitude at high order harmonics of the stimulation pulses proportionately modulates corticospinal contribution that is being indexed by the normalized additional force (Δ*F*%).

**Figure 6:**
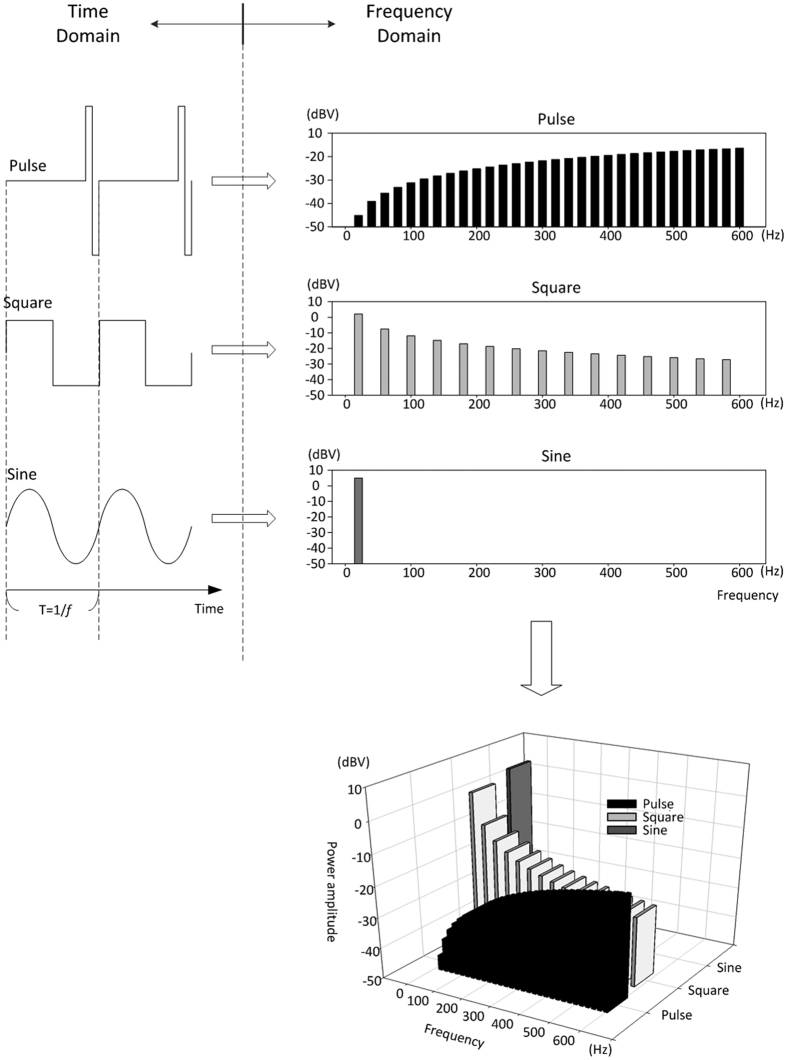
Harmonic analysis of pulse (pulse width = 250 μs, biphasic), square, and sine functions applied at the same frequency (20 Hz) but normalized intensities. Intensity of the square function is 1 Ampere, whereas intensities of pulse and sine functions are normalized such that the pulse, square, and sine functions carry the same amount of electrical energy during the same period of time. Please refer to the Supplementary File for a more detailed description of the harmonic analysis.

Sinusoidal, square, and pulse functions can demonstrate different distributions of power harmonics in their respective frequency spectrums (Fig. 6). As illustrated by the diagrams in Fig. 6, when the different stimulation waveforms carry the same amount of electrical energy during the same period of time, the pulse function provides additional power at higher frequency bandwidths (i.e., as denoted by the greater amplitudes of power harmonics between 100–600 Hz in Fig. 6), unlike sinusoidal or square functions, which generate no harmonics or only harmonics of decreasing amplitude at higher frequency bandwidths. As the change of ion channel permeability and membrane properties depend on the power at different frequency bandwidths^31^,^32^,^33^, we speculate that the change of corticospinal effects may be caused by the change of ion channel permeability or membrane properties of the stimulated afferent neural pathway, which may have a modulation effect on the corticospinal contribution of force enhancements. The results of this study are consistent with several previous studies that focus on the stimulation frequency factor as well as studies that examine the role of pulse duration^34^,^35^,^36^,^37^,^38^, likely due to the fact that both stimulation frequency and pulse duration can alter the distribution of harmonics in a frequency spectrum. This underscores the potential for establishing an ideal stimulation frequency pass band for peripheral nerve stimulation applications, with the objective of selectively activating the occurrence of neuroplasticity at the CNS.

Tremendous efforts have been made on the engineering and clinical validation front to advance the science and efficacy of electrical stimulation. Stimulation waveform, along with anatomical target and clinical/demographic factors, are fundamental parameters in such studies. In this study, we expand on the role of waveform by introducing a novel concept: although it is known that stimulation frequency of pulses is important (when the “frequency” of stimulation is reported, this typically indicates the timing of pulses and not the signal frequency content), it is also important to consider high-order power (frequency content in harmonics across the spectrum). Indeed, these results may change our interpretation of low vs. high pulse frequency stimulation, as low-frequency with harmonics may have similar harmonic components as high-frequency stimulation^39^,^40^,^41^,^42^,^43^. Conversely, these results may impact very high (kHz) pulse frequency stimulation where sub-harmonics may be important, noting that pulse-shapes influence harmonics^44^,^45^,^46^,^47^,^48^. In addition, the novel concept of stimulation by power harmonics may also inform the results of stimulation using varied pulse frequencies, where additional harmonic content is generated^49^,^50^,^51^,^52^.

In the course of this study, we did not attempt to collect additional data to prove that a higher-intensity stimulation train induced more additional force than a lower-intensity stimulation train as this has been demonstrated in an earlier study^14^. Although this may limit our ability to measure the extent of priming effects in consecutive electrical stimulation trains and the correlation between the power harmonics and the corticospinal effects specifically relevant to the priming effects, we believe this does not detract from the main objective of this study, which is to find the relationship between the high frequency components (higher order harmonics) and the observed corticospinal effects relevant to the same train of electrical stimulation.

## Additional Information

**How to cite this article**: Chen, C.-F. *et al*. Higher-order power harmonics of pulsed electrical stimulation modulates corticospinal contribution of peripheral nerve stimulation. *Sci. Rep.* **7**, 43619; doi: 10.1038/srep43619 (2017).

**Publisher's note:** Springer Nature remains neutral with regard to jurisdictional claims in published maps and institutional affiliations.

## Supplementary Material

Supplementary Information

## Figures and Tables

**Table 1:** The correlation coefficient (*r*) of INT/CTRL ratios of normalized additional force Δ*F*% (Δ*F*_INT_%/Δ*F*_CTRL_%) and harmonic amplitude (dB V_INT_/V_CTRL_) in different frequency bands.

	Correlation Coefficient (*r*)	
20–80 Hz	*r* = 0.4247^a^	(*p* = 0.0243, n = 28)^a^
100–180 Hz	*r* = 0.5894	(*p* = 0.0001, n = 40)
200–280 Hz	*r* = 0.7002	(*p* < 0.0001, n = 40)
300–380 Hz	*r* = 0.7449	(*p* < 0.0001, n = 40)
400–480 Hz	*r* = 0.7906	(*p* < 0.0001, n = 40)
500–600 Hz	*r* = 0.7717	(*p* < 0.0001, n = 48)

^a^Outliers have been removed based on postestimation diagnostic^53^. Sample points that fall in the region of X + Y > 0.06 (Y: Leverage, X: Normalized residual squared) were considered as outliers. Before removing outliers, *r* = 0.2594 (*p* = 0.1516, n = 32) in the 20–80 Hz frequency range.

**Table 2:** Statistical comparisons^b^ between the correlation coefficients (20–80 Hz vs. higher frequency bands): A higher z score indicates greater difference between the correlation coefficients and *p* < 0.05 indicates a level of statistical significance.

	*z* score and *p* value	
20–80 Hz vs. 100–180 Hz	*z* = −0.863	*p* = 0.1942
20–80 Hz vs. 200–280 Hz	*z* = −1.600	*p* = 0.0548
20–80 Hz vs. 300–380 Hz	*z* = −1.962	*p* = 0.0249
20–80 Hz vs. 400–480 Hz	*z* = −2.393	*p* = 0.0084
20–80 Hz vs. 500–600 Hz	*z* = −2.290	*p* = 0.0110

^b^Results were provided by Stata’s cortesti command, one-tailed test was applied.
