# Supplementary material for: Higher-order power harmonics of pulsed electrical stimulation modulates corticospinal contribution of peripheral nerve stimulation
Source: Sci Rep. 2017 Mar 3;7:43619. doi: 10.1038/srep43619 (PMC5335254; doi:10.1038/srep43619)
Supplement: Supplementary Information [file srep43619-s1.pdf]

Higher-order power harmonics of pulsed electrical stimulation modulates corticospinal contribution of peripheral nerve stimulation

– Supplementary description of the power spectrums in Fig. 6

Chiun-Fan Chen[1], Marom Bikson[2], Li-Wei Chou[3], Chunlei Shan[1,4], Niranjan Khadka[2], Wen-Shiang Chen[5], Felipe Fregni*[1]

[1]Spaulding Neuromodulation Center, Department of Physical Medicine & Rehabilitation, Spaulding Rehabilitation Hospital and Massachusetts General Hospital, Harvard Medical School, Boston, MA, USA

[2]Department of Biomedical Engineering, The City College of the City University of New York, NY, USA

[3]Department of Physical Therapy and Assistive Technologies, National Yang-Ming University, Taipei, Taiwan

[4]School of Rehabilitation Science, Shanghai University of Traditional Chinese Medicine, Shanghai, China

[5]Department of Physical Medicine and Rehabilitation, National Taiwan University College of Medicine and National Taiwan University Hospital

*Corresponding Author

Supplementary description of the power spectrums in Fig. 6

The Fourier series of a periodic function f(t) is the expansion of f(t) into a collection of sinusoidal functions (sine and cosine functions) having the same frequency.

$$f(t) = a_0 + \sum_{n=1}^{\infty} a_n \cos(2\pi f tn) + \sum_{n=1}^{\infty} b_n \sin(2\pi f tn)$$

, wherein *f* is the frequency of f(t), and a_0, a_n, and b_n are coefficients that can be derived as follows (T is the period of f(t), therefore T=1/*f*):

$$a_0 = \frac{1}{T}\int_{-T/2}^{T/2} f(t)dt$$

$$a_n = \frac{2}{T}\int_{-T/2}^{T/2} f(t)\cos\left(\frac{2\pi tn}{T}\right)dt$$

$$b_n = \frac{2}{T}\int_{-T/2}^{T/2} f(t)\sin\left(\frac{2\pi tn}{T}\right)dt$$

The derived coefficients a_0, a_n, and b_n are used to construct the frequency spectrum (or power spectrum) of f(t) by calculating the power amplitude at all frequencies, where a_0 is the amplitude at DC (direct current, zero frequency) and $\sqrt{a_n^2+b_n^2}$ is the amplitude at all other frequencies.

The following is the derivation of Fourier series for the **pulse** p(t), **square** q(t) and **sine** r(t) functions that construct the power spectrums in Fig. 6:

1. p(t) is a **pulse** function of frequency *f* = 20Hz, period T = 1/*f* = 50ms, pulse width w = 250µs, and intensity A.

$$p(t) = a_0 + \sum_{n=1}^{\infty} a_n \cos(2\pi f tn) + \sum_{n=1}^{\infty} b_n \sin(2\pi f tn)$$

Derivation of coefficients a_0, a_n, and b_n:

$$a_0 = \frac{1}{T}\int_{-T/2}^{T/2} p(t)dt = 0$$

$$a_n = \frac{2}{T}\int_{-T/2}^{T/2} p(t)\cos\left(\frac{2\pi tn}{T}\right)dt$$

$$= \frac{2}{T}\left[\int_{-9w/8}^{-w/8} -A\cos\left(\frac{2\pi tn}{T}\right)dt + \int_{w/8}^{9w/8} A\cos\left(\frac{2\pi tn}{T}\right)dt\right]$$

$$= \frac{2A}{T}\frac{T}{2\pi n}\left[-\sin\left(\frac{2\pi tn}{T}\right)\Big|_{-9w/8}^{-w/8} + \sin\left(\frac{2\pi tn}{T}\right)\Big|_{w/8}^{9w/8}\right] = 0$$

$$b_n = \frac{2}{T}\int_{-T/2}^{T/2} p(t)\sin\left(\frac{2\pi tn}{T}\right)dt$$

$$= \frac{2}{T}\left[\int_{-9w/8}^{-w/8} -A\sin\left(\frac{2\pi tn}{T}\right)dt + \int_{w/8}^{9w/8} A\sin\left(\frac{2\pi tn}{T}\right)dt\right]$$

$$= \frac{2A}{T}\frac{T}{2\pi n}\left[\cos\left(\frac{2\pi tn}{T}\right)|_{-9w/8}^{-w/8} - \cos\left(\frac{2\pi tn}{T}\right)|_{w/8}^{9w/8}\right]$$

$$= \frac{A}{\pi n}\left[2\cos\left(\frac{2\pi tn}{T}\right)|_{t=w/8} - 2\cos\left(\frac{2\pi tn}{T}\right)|_{t=9w/8}\right]$$

$$= \frac{2A}{\pi n}\left[\cos\left(\frac{\pi wn}{4T}\right) - \cos\left(\frac{9\pi wn}{4T}\right)\right]$$

Fourier series of the **pulse** function p(t):

$$p(t) = \sum_{n=1}^{\infty} b_n \sin(2\pi f tn) = \sum_{n=1}^{\infty}\frac{2A}{\pi n}\left[\cos\left(\frac{\pi wn}{4T}\right) - \cos\left(\frac{9\pi wn}{4T}\right)\right]\sin(2\pi f tn)$$

$$= \sum_{n=1}^{\infty}\frac{2A}{\pi n}\left[\cos\left(\frac{\pi n}{800}\right) - \cos\left(\frac{9\pi n}{800}\right)\right]\sin(40\pi tn)$$

$$, \text{where } \frac{w}{T} = \frac{0.250\text{ms}}{50\text{ms}} = \frac{250}{50000} = \frac{1}{200}$$

The following are the amplitudes in the power spectrum of the **pulse** function as shown in Fig. 6, where A_{pulse} is the normalized A ($A_{pulse} = A_{square} \times\sqrt{200}$) such that the pulse, square, and sine functions carry the same amount of electrical energy during the same period of time[1]:

$$20\log b_n = 20\log\frac{2A_{pulse}}{\pi n}\left[\cos\left(\frac{\pi n}{800}\right) - \cos\left(\frac{9\pi n}{800}\right)\right], n \times 20\text{Hz}$$

$$20\log b_1 = 20\log\frac{2A_{pulse}}{\pi}\left[\cos\left(\frac{\pi}{800}\right) - \cos\left(\frac{9\pi}{800}\right)\right], 20\text{Hz}$$

$$20\log b_2 = 20\log\frac{2A_{pulse}}{2\pi}\left[\cos\left(\frac{\pi}{800}\right) - \cos\left(\frac{9\pi}{800}\right)\right], 40\text{Hz}$$

$$20\log b_3 = 20\log\frac{2A_{pulse}}{3\pi}\left[\cos\left(\frac{\pi}{800}\right) - \cos\left(\frac{9\pi}{800}\right)\right], 60\text{Hz}$$

....

2. <u>q(t) is a **square** function of frequency *f* = 20Hz, period T = 1/*f* = 50ms, and</u>

---

[1] $\int_0^T [A_{pulse}\, p(t)]^2 dt = \int_0^T [A_{square}\, q(t)]^2 dt = \int_0^T [A_{sine}\, r(t)]^2 dt$, under the assumption that impedance Z is a constant value

intensity A.

$$q(t) = a_0 + \sum_{n=1}^{\infty} a_n \cos(2\pi f tn) + \sum_{n=1}^{\infty} b_n \sin(2\pi f tn)$$

Derivation of coefficients a_0, a_n, and b_n:

$$a_0 = \frac{1}{T}\int_{-T/2}^{T/2} q(t)dt = 0$$

$$a_n = \frac{2}{T}\int_{-T/2}^{T/2} q(t)\cos\left(\frac{2\pi tn}{T}\right)dt$$

$$= \frac{2}{T}\left[\int_{-T/2}^{0} -A\cos\left(\frac{2\pi tn}{T}\right)dt + \int_{0}^{T/2} A\cos\left(\frac{2\pi tn}{T}\right)dt\right]$$

$$= \frac{2A}{T}\frac{T}{2\pi n}\left[-\sin\left(\frac{2\pi tn}{T}\right)|_{-T/2}^{0} + \sin\left(\frac{2\pi tn}{T}\right)|_{0}^{T/2}\right] = 0$$

$$b_n = \frac{2}{T}\int_{-T/2}^{T/2} q(t)\sin\left(\frac{2\pi tn}{T}\right)dt$$

$$= \frac{2}{T}\left[\int_{-T/2}^{0} -A\sin\left(\frac{2\pi tn}{T}\right)dt + \int_{0}^{T/2} A\sin\left(\frac{2\pi tn}{T}\right)dt\right]$$

$$= \frac{2A}{T}\frac{T}{2\pi n}\left[\cos\left(\frac{2\pi tn}{T}\right)|_{-T/2}^{0} - \cos\left(\frac{2\pi tn}{T}\right)|_{0}^{T/2}\right]$$

$$= \frac{A}{\pi n}[1 - \cos(\pi n) - \cos(\pi n) + 1] = \frac{2A}{\pi n}[1 - \cos(\pi n)]$$

Fourier series of the **square** function q(t):

$$q(t) = \sum_{n=1}^{\infty} b_n \sin(2\pi f tn) = \sum_{n=1}^{\infty} \frac{2A}{\pi n}[1 - \cos(\pi n)]\sin(40\pi tn)$$

The following are the amplitudes in the power spectrum of the **square** function as shown in Fig. 6:

$$20\log b_n = 20\log\frac{2A_{square}}{\pi n}[1 - \cos(\pi n)], n \times 20\text{Hz}$$

$$20\log b_1 = 20\log\frac{2A_{square}}{\pi}[1 - \cos(\pi)], 20\text{Hz}$$

$$20\log b_2 = 20\log\frac{2A_{square}}{2\pi}[1 - \cos(2\pi)], 40\text{Hz}$$

$$20\log b_3 = 20\log\frac{2A_{square}}{3\pi}[1 - \cos(3\pi)], 60\text{Hz}$$

....

3. r(t) is a sine function of frequency f = 20Hz, period T = 1/f = 50ms, and intensity A.

$$p(t) = a_0 + \sum_{n=1}^{\infty} a_n \cos(2\pi f tn) + \sum_{n=1}^{\infty} b_n \sin(2\pi f tn) = \sin(40\pi tn)$$

The following is the amplitude in the power spectrum of the **sine** function as shown in Fig. 6, wherein A_{sine} is the normalized A ($A_{sine} = A_{square} \times \sqrt{\pi}$) such that the pulse, square, and sine functions carry the same amount of electrical energy during the same period of time[2]:

$$20\log b_1 = 20\log\frac{2A_{sine}}{\pi}[1 - \cos(\pi)], 20Hz$$

(no value at other frequencies)

References

1. Oppenheim A, Willsky A, Hamid S: *Signals and systems.* 2nd edn. Upper Saddle River, NJ: Prentice Hall; 1983.

2. Oppenheim A, Schafer R, Buck J: *Discrete-time signal processing.* 2nd edn. Upper Saddle River, NJ: Prentice Hall; 1999.

3. Weisstein, Eric W. "Fourier Series." From *MathWorld*--A Wolfram Web Resource. http://mathworld.wolfram.com/FourierSeries.html

4. Weisstein, Eric W. "Fourier Transform." From *MathWorld*--A Wolfram Web Resource. http://mathworld.wolfram.com/FourierTransform.html

[2] $\int_0^T [A_{pulse}\, p(t)]^2 dt = \int_0^T [A_{square}\, q(t)]^2 dt = \int_0^T [A_{sine}\, r(t)]^2 dt$, under the assumption that impedance Z is a constant value
